# Predictors of the use and approval of CAM: results from the German General Social Survey (ALLBUS)

**DOI:** 10.1186/s12906-020-02966-9

**Published:** 2020-06-11

**Authors:** Henrik Abheiden, Michael Teut, Anne Berghöfer

**Affiliations:** Institute for Social Medicine, Epidemiology and Health Economics, Charité – Universitätsmedizin Berlin, Luisenstraße 57, 10117 Berlin, Germany

**Keywords:** Complementary medicine, Alternative medicine, Health care utilization, Attitude, Paranormal beliefs, Education, Female, ALLBUS, Cross-sectional survey, Germany

## Abstract

**Background:**

Many studies have shown that sociodemographic variables significantly predict the use of complementary and alternative medicine (CAM), although these predictions were not particularly strong. A multitude of predictors of the use or approval of CAM have been investigated in the field of personal values and worldviews, but the effects were small or doubtful due to non-representative samples. More recent psychological research has linked positive attitudes towards CAM with intuitive thinking, paranormal beliefs, ontological confusions and magical health beliefs, suggesting a common thinking style behind all these variables. The aim of this study is to identify the most important predictors of the use and approval of CAM.

**Methods:**

We performed a canonical correlation analysis on all 3480 records from the 2012 German General Social Survey (ALLBUS) with the lifetime use and opinion of CAM as the dependent variables.

**Results:**

Approval of paranormal practices such as fortune-telling, dowsing or spiritualism explained 32% of the variance in the dependent canonical variate “approval of CAM”, while sociodemographic variables explained only 2%. Experience with paranormal practices explained 17% of the variance in the dependent canonical variate “experience with CAM”, and sociodemographic variables explained 10% of the variance. Traditional religiosity, attitudes towards science and post-materialist values showed no relevant correlations with the dependent canonical variates.

**Conclusions:**

Paranormal beliefs and related measures are the most important known predictors of the use and approval of CAM. Experience with paranormal practices not only indicates paranormal beliefs but also explains experience with CAM that cannot be explained by approval of CAM. Female gender and higher socioeconomic status predict experience with CAM without predicting approval of CAM, but their influence should not be overstated.

## Background

According to a classical definition, complementary and alternative medicine (CAM) refers to medical interventions that are not taught widely at medical schools and are not generally available at hospitals [1]. However, this definition is partly outdated. Some CAM therapies are part of public health care in some countries because of tradition or proven effectiveness. Examples of CAM include: herbal medicine, traditional European medicine, traditional Chinese medicine (including acupuncture and Chinese herbology), traditional Indian medicine/Ayurveda, homeopathy, anthroposophic medicine, osteopathy/chiropractic, energy medicine (including spiritual healing, distant healing, reiki), mind-body medicine (including lifestyle advice, relaxation, meditation, mindfulness, hypnotherapy), exercise (including yoga, qigong), nutritional therapy.

The use of CAM is common in Germany. Incidence estimates range between 40 and 62% for past year use and 63 and 76% for lifetime use [2]. As in many other Western countries, female gender, middle age and higher educational level have been shown to predict CAM use; some papers report general socioeconomic status instead of education, and some report income as an additional positive predictor [3–7]. Illness predicts the use of both conventional medicine and CAM, which might explain why the use of conventional medicine and the use of CAM are positively correlated [8].

The term “alternative medicine” suggests that CAM users are primarily motivated by the rejection of conventional medicine. International research has painted a different picture. Disappointment with conventional medicine predicts neither the use [9] nor the approval [10–12] of CAM. Instead, most CAM users combine CAM and conventional medicine pragmatically; only a small portion of users rely primarily or solely on CAM [9, 13]. Opposition to conventional medicine might be a motivation among this subgroup [9].

In the late twentieth century, the spreading of New Age beliefs (in particular, “holistic” attitudes towards health) or postmodern values (including sentiments against science and technology, idealization of nature and demand for participation in medical decisions) was a popular explanation for the ongoing rise in CAM use. Many studies yielded results supporting that idea, but the effects were usually small [9], the samples were not sufficiently representative of the general population [14] or both [15–18]. Two studies require special attention. In 1998, Astin concluded: “the majority of alternative medicine users appear to be doing so [ …] largely because they find these health care alternatives to be more congruent with their own values, beliefs, and philosophical orientations toward health and life” [9]. Although cited frequently, this interpretation, particularly the word “largely”, is not justified by the effect sizes: the largest correlation found with CAM use was 0.17 (for being a “cultural creative”), which corresponds to an estimated risk ratio of 1.5. The paper instead provides an odds ratio of 1.95 as the result of a multiple logistic regression. Note that odds ratios are always expressed in more extreme numbers than the corresponding risk ratios, especially if the dependent variable represents something as common as CAM use. In the second study, postmodern values were shown to explain 20% of the variance in attitudes towards CAM in the last step of a hierarchical regression analysis [10]. The share of explained variance, also known as R^2^, is an excellent measure of predictive relevance. If a variable or a group of variables predicts the dependent variable perfectly, this corresponds to 100% explained variance. If a predictor explains 20% of the variance, then only four other equally strong independent predictors could possibly exist. Random measurement errors in dependent or independent variables cause the share of explained variance to be underestimated. Furthermore, attitudes towards CAM are certainly affected by a multitude of personal reasons that a statistical model will never be able to account for. Considering all this, 20% explained variance denotes a good predictor of attitudes towards CAM. In that study, however, a closer look at the regression coefficients reveals that neither anti-science sentiments, holism, rejection of authority nor individual responsibility strongly contributed to the prediction. Instead, a preference for natural remedies and an appreciation of a variety of therapies to choose from predicted approval of CAM; both “predictors” are in part only a measure of approval of CAM itself. The previous step of the hierarchical regression included sociodemographic variables and attitudes towards conventional medicine alone, which together accounted for 4% of the variance.

In a Swiss study, post-materialist values (or rather, the absence of materialist values, odds ratio 0.6) and neo-religious beliefs (odds ratio 1.7) predicted CAM use, whereas traditional Christian beliefs slightly predicted non-use (odds ratio 0.8) [19]. The regressors were taken from two factor analyses; the factor interpreted as neo-religious beliefs mainly captured reincarnation and other non-Christian ideas about existence after death. These results might correspond to the finding that self-rated spirituality predicts use (odds ratio 1.58, standardized on the reported standard deviation), while self-rated religiosity does not [8]. Not disagreeing with spiritual experience as the source of the most important knowledge was shown to more than double the odds of using CAM [20].

A new approach originated from psychological research on students: intuitive thinking, paranormal beliefs and magical food and health beliefs accounted for 28% of the variance in CAM belief [21]. All these concepts were positively correlated with one another and weakly correlated with being female, thus fully explaining gender differences in CAM belief. No correlation was found between rational thinking and belief in CAM. Paranormal beliefs were measured with a 26-item questionnaire covering traditional religious belief, psi, witchcraft, superstition, spiritualism, extraordinary life forms and precognition. The authors conceptualized magical beliefs based on J. G. Frazer’s two laws of magical thinking [22]: the law of contagion states that characteristics of one object or person can be transferred to another object or person by physical contact, and the law of similarity says that superficial resemblance indicates or causes deep resemblance. The authors argue that these laws are also present in varieties of CAM, such as homeopathy, detoxification or several forms of energy medicine. The terms rational and intuitive thinking refer to dual process theories, according to which humans have two modes of processing information: rational thinking is slow and exhausting; it allows conscious, logical conclusions but is easily overwhelmed by complexity. Intuitive thinking, on the other hand, is fast, automatic, associative and mainly unconscious; it handles complexity by drawing on experience and heuristics. Intuitive judgements solidify over time and are hard to correct, unlike rational judgements, which can change dramatically in light of new information. The authors concluded that it was not disability of rational thinking but rather the tendency to employ intuitive instead of rational thinking that predisposed people to approve of CAM. A subsequent study on a cross section of the Finnish population aged 15 to 56 found that intuitive thinking, paranormal beliefs and ontological confusions explained 34% of the variance in CAM belief [12]. Ontological confusion refers to the tendency to transfer properties of animate things to inanimate things, or vice versa. Additional variables accounted for a further 4% of the variance in CAM belief: desire for control in medical decisions and self-characterization as an environmentalist contributed to this model expansion; gender, education, income, age, health and self-attribution to an unconventional, feminist, exotic or natural worldview did not. A Flemish cross-sectional study confirmed that paranormal beliefs predicted CAM belief and explained 14% of the variance [23]. Age explained another 3% as a positive predictor; education, gender and attitudes towards science and technology did not predict belief in CAM. Cross-sectional data from an Australian online panel provided further evidence that magical health beliefs were an important predictor of positive attitudes towards CAM; holistic health beliefs did not contribute to the prediction [24]. Magical health beliefs, CAM attitudes and vaccination scepticism were positively intercorrelated.

Finally, there is some evidence that absorption (i.e., the tendency to become absorbed in mental imagery, a personality trait related to openness to experience), internal locus of control and various coping styles predict CAM use or CAM belief, but the effects tend to be small, inconsistent and doubtful due to the number or quality of studies [25]. Openness to experience appears to be a rather consistent predictor of use but not of approval [25–27].

Research has not yet demonstrated clear differences between predictors of use and predictors of approval of CAM. Sociodemographic variables seem to be more important for the use of CAM than for the opinion of CAM; they do not predict trust in CAM either [11]. Considering that use and approval are positively correlated [24], similar predictors are somewhat expectable. It is not known how the correlation between use and approval is mediated. Approval is presumably a reason for use. On the other hand, users tend to report a higher opinion of CAM as a result of experience in retrospect [4].

In summary, research on predictors of the use and approval of CAM can roughly be divided into three fields: sociodemographics, personal values and worldviews, and psychological measures related to paranormal beliefs. In this study, we include variables from all three fields to identify the most important predictors based on a large cross-sectional sample. Another aim is to distinguish predictors of approval of CAM from predictors of experience with CAM. To our knowledge, the role of paranormal practices in the prediction of CAM *use* has not been investigated before.

## Methods

### Sampling and data collection

The German General Social Survey (ALLBUS) is a programme designed to produce high-quality data for the scientific public [28]. Since 1980, every 2 years, a new cross-sectional survey has been carried out with a core set of permanent variables and varying focus topics, which are normally repeated every 10 years. For our analysis, we use all 3480 records from the 2012 survey, which focussed on religion and worldview and included questions on CAM as an aspect of personal beliefs.

A two-step sampling design and elaborate fieldwork make the sample representative of the residents of Germany born before 1994. In the first step of the sampling process, 162 sample points were randomly selected; in the second step, participants were sampled from the local resident registers stratified by age and gender. The former territory of East Germany has been deliberately oversampled, which we compensate for by weighting records, as proposed by the ALLBUS authors. The selected people were visited for computer-assisted personal interviews, which lasted 54 min on average. Respondents were compensated with 10 euros. Thirty-eight percent of the planned interviews could be completed.

### Handling of missing data

We impute missing data by running an iterative regression algorithm on the set of all used variables; random error is added to the imputed values as demanded by residual variance [29, 30]. The choice of a specific method of imputation is in fact of little importance to the results, as missing data are rare in most variables. Household income forms an exception because 13.6% of the interviewees refused to answer. Another 14.5% categorized their household income into one of 22 ranges instead of providing an exact number. In these cases, we imputed the geometric mean of the given exact values falling in the same range.

### Analysis

All results are generated by a single canonical correlation analysis (CCA). CCA is a classical multivariate method, meaning it analyses more than one dependent variable at a time. We chose a multivariate analysis to reveal commonalities and differences in the predictors of lifetime use and approval of different CAM practices and to consider the interaction between lifetime use and approval. CCA can be used to analyse metric and categorical variables (expressed as dummy variables). The variables do not have to follow specific distributions as long as no test is to be performed. Many other methods, such as multiple linear regression, linear discriminant analysis or even the χ^2^ test of independence, are special cases of CCA. The interpretation of a CCA is similar to that of a factor analysis. For those who are not familiar with factor analysis, the following paragraph provides an explanation based on multiple linear regression.

In multiple regression analysis, a new variable is calculated as a linear combination (i.e., weighted sum) of the independent variables. The weights are chosen to maximize the correlation between the new variable and the dependent variable. CCA replaces the single dependent variable with a linear combination of dependent variables. Maximizing the correlation between the two new variables (in CCA terminology: canonical variates) remains the criterion that defines the weights of the variables. For an unambiguous solution, canonical variates have a variance of 1 by definition; in addition, we have mean-centred the canonical variates. Further pairs of canonical variates with decreasing correlations can be calculated so that they are uncorrelated with all other canonical variates. The correlation of the n^th^ pair of canonical variates is called the n^th^ canonical correlation. Theoretically, there are as many pairs as there are variables in the smaller set. The relevant information, however, is concentrated in the first pairs. Thus, CCA reduces the complexity of the relationships between the variables to a few dimensions.

### Presentation of results

CCA itself treats both sets of variables equally. For better comprehensibility, we nevertheless refer to the four variables on CAM as “dependent” and present the results accordingly. The interviewees were asked if they had personal experience with and what they thought of “miracle healers/spirit healers”, “yoga/tai chi/qigong”, “medicines of the Far East: Ayurveda, reiki, shiatsu, and the like” and “other alternative medicines: homoeopathy, Bach flower therapy, and the like” (F032, F033). For interviewees who expressed that they had never heard of or had no opinion about an item, we created the category “no opinion”. The same questions were asked for “magic/spiritism/occultism”, “pendulum dowsing/divining”, “astrology/horoscopes” and “tarot cards/fortune telling”. We refer to these as “paranormal practices” and include the corresponding variables in the set of independent variables. The ALLBUS authors provided detailed documentation, including an English translation of the questionnaire [28]. Therefore, instead of describing every variable here, we give the underlying questions (named F or S followed by 3 digits) in the results section.

To make the weights of metric variables comparable with each other, they are standardized by multiplying them by the standard deviation of the variable. The weights of dichotomous or dummy variables are contrasted against an average person instead of a selected reference level. As the weights of intercorrelated variables can be hard to interpret, we base our interpretation primarily on correlations between variables and dependent canonical variates. Correlations of variables with canonical variates built from the same set of variables are called canonical loadings; they render canonical variates a meaning similar to factor loadings in factor analysis. Correlations of variables with canonical variates built from the other set of variables are known as canonical cross-loadings; they indicate the relevance of predictors and can be calculated from canonical loadings by simply multiplying with the respective canonical correlation. For dichotomous or dummy variables, we report the means of the dependent canonical variates within the respective groups of interviewees instead of correlations. To obtain a single and uniform measure of loading or predictive relevance for both metric and non-metric variables, we calculate the share of variance explained in the dependent canonical variates for every variable (as the squared multiple correlation). The same is done for the groups of sociodemographic variables and paranormal practices to assess their redundancy as predictors.

## Results

We report the first three dimensions of the model, which we believe include all the relevant information. The unabridged 24-dimensional model is included in additional file 1.

### Dependent canonical variates

**Fig. 1:**
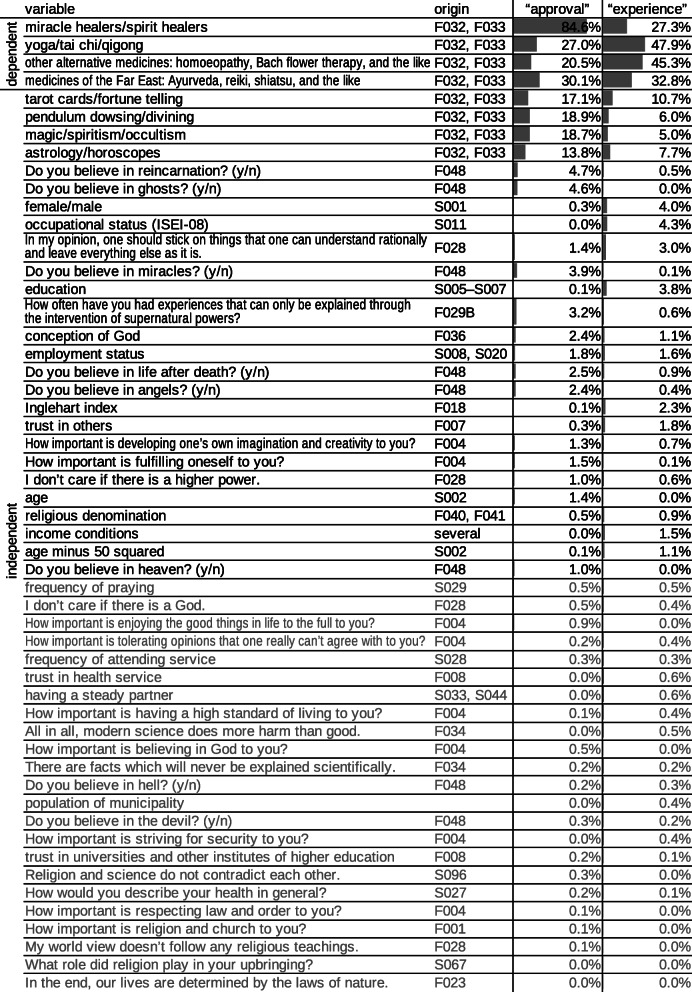
Share of variance explained in the dependent canonical variates “approval” and “experience” in descending order of sum. Variables that explain a total of less than 1% variance are marked in gray

**Fig. 2:**
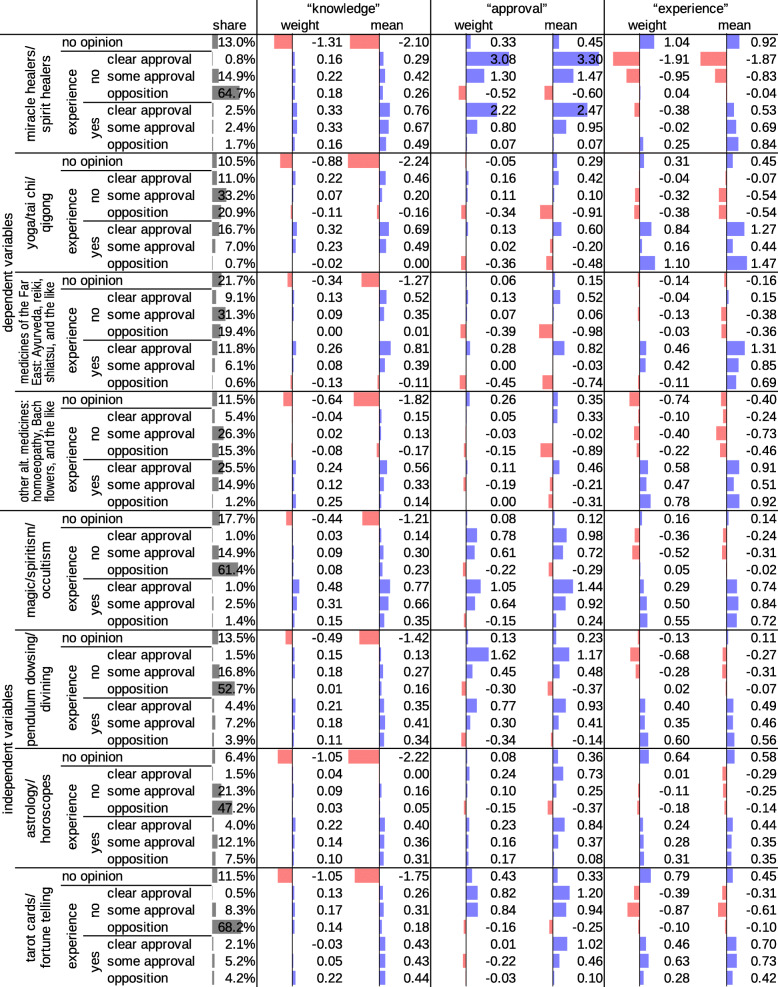
Representation of CAM in the dependent canonical variates and predictions by paranormal practices: Weights for the calculation of the canonical variates and means in the dependent canonical variates

The first dependent canonical variate is a measure of CAM knowledge: people who do not know many varieties of CAM or do not have an opinion about many varieties of CAM have low scores on the first dependent canonical variate (Fig. 2). The second and third dependent canonical variates represent approval of CAM and experience with CAM. In the raw model, these concepts are somewhat mixed. To increase interpretability, we combined and contrasted the original dependent canonical variates two and three in a way that mathematically corresponds to a rotation by 30 degrees. We refer to these rotated dependent canonical variates as “approval” and “experience”, respectively. People reach high scores on “approval” if they approve of many varieties of CAM, especially if they clearly approve (Fig. 2). People reach high scores on “experience” if they have tried many varieties of CAM (Fig. 2). The top part of Fig. 1 shows how well the different varieties of CAM are represented in the variates “approval” and “experience”. In “approval”, spiritual healing is represented best, which means that the model is better at predicting approval of spiritual healing than at predicting approval of other varieties of CAM. In “experience”, however, spiritual healing is less represented than the other three varieties of CAM, and therefore, predictions of experience with spiritual healing are less accurate.

### Predictors

**Fig. 3:**
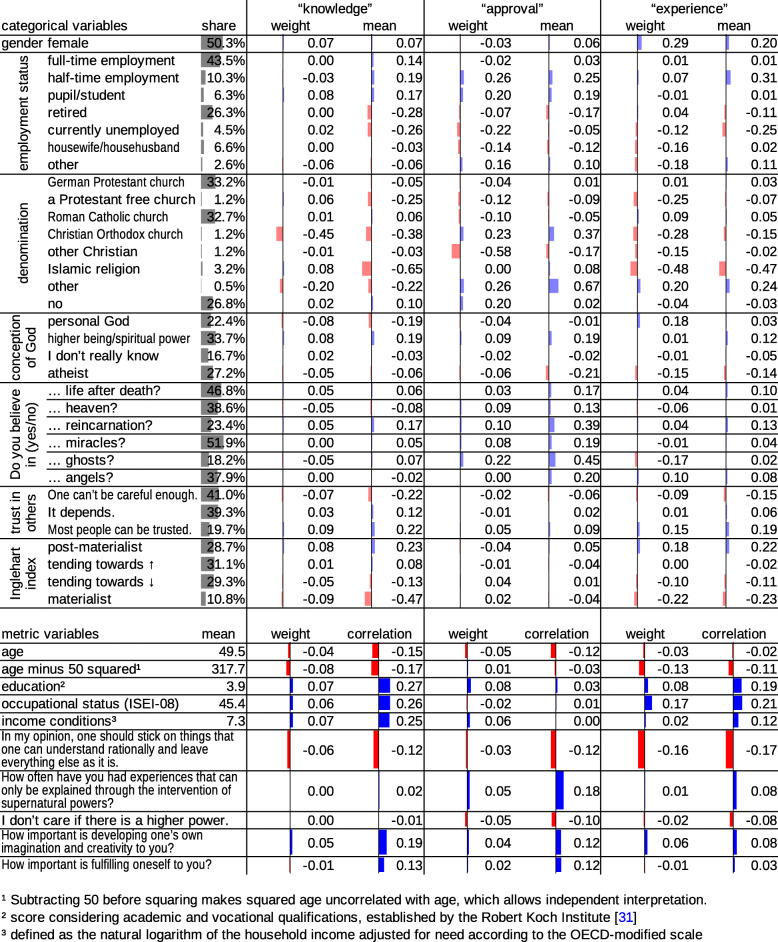
Predictions by the other independent variables: Weights for the calculation of the canonical variates (standardized in case of metric variables) and means in or correlations with the dependent canonical variates

The bottom part of Fig. 1 ranks the independent variables by how well they explain “approval” and “experience”. Figures 2 and 3 give a detailed account of how the independent variables contribute to the prediction.

Twenty-three independent variables were completely irrelevant and therefore omitted from Figs. 2 and 3. Each of them explained a total of less than 1% of the variance in “approval” and “experience” (Fig. 1). Among these variables are most questions on traditional religiosity, all questions on science and academic medicine and most questions on important things in life. We also did not find any effect for having a steady partner, for the population of the municipality or for self-rated health.

Explaining 56% of the variance in the first dependent canonical variate, the best predictors for knowing and having an opinion about CAM are knowing and having an opinion about paranormal practices (Fig. 1, Fig. 2). Sociodemographic variables (i.e., gender, age, squared age, education, occupational status, income conditions and employment status) explain 15% of the variance, with higher socioeconomic status and young or middle age as the strongest predictors (Fig. 1, Fig. 3). Predictions by sociodemographic variables are largely redundant with predictions by paranormal practices: The combination of both sets of variables explains 60% of the variance, which is much less than the sum of 56 and 15%.

Approval of CAM is best predicted by approval of paranormal practices (Fig. 1, Fig. 2). The four main variables of paranormal practices explain 32% of the variance in “approval”. Weaker predictors that also represent paranormal or supernatural beliefs include belief in reincarnation, ghosts, miracles, life after death and angels (F048), belief in “some kind of higher being or a spiritual power” (as opposed to belief in a personal or no God at all, F036) and the frequency of having “had experiences that can only be explained through the intervention of supernatural powers” (F029B) (Fig. 1, Fig. 3). Explaining only 2% variance, sociodemographic variables are basically irrelevant in the prediction of approval of CAM (Fig. 1, Fig. 3).

Experience with CAM is best predicted by experience with paranormal practices (Fig. 1, Fig. 2). The four main variables of paranormal practices explain 17% of the variance in “experience”. Sociodemographic variables explain 10% of the variance largely nonredundantly (the combination of both sets of variables explains 26% of the variance). Among the sociodemographic variables, female gender, higher occupational status and higher educational level are the strongest predictors (Fig. 1, Fig. 3). With a weight close to zero, income conditions cannot be considered an independent predictor (Fig. 3). Nevertheless, income conditions slightly correlate with “experience”. This phenomenon can be explained by confounding: income conditions correlate with “experience” because they are a measure of general socioeconomic status (alongside education and occupation). Opposition to the statement “In my opinion, one should stick on things that one can understand rationally and leave everything else as it is” (F028) and post-materialism, as measured by the Inglehart index (F018) [32], are minor predictors of experience with CAM (Fig. 1, Fig. 3).

## Discussion

### Main results

In our sample, lifetime use and approval of paranormal practices such as fortune-telling, dowsing or spiritualism are by far the best predictors of the lifetime use and approval of CAM. Sociodemographic variables explain some additional variance in use, with female gender and general socioeconomic status as the strongest predictors. Income conditions, traditional religiosity and attitudes towards science predict neither the use nor the approval of CAM. The CAM practices included in this analysis are intercorrelated in both use and approval; we did not find differences in predictors.

### Relation to previous findings

Our results regarding paranormal practices and sociodemographic variables confirm previous findings and create a more coherent overall picture. The existing psychological research has linked positive attitudes towards CAM with intuitive thinking, paranormal beliefs, ontological confusions and magical health beliefs, suggesting a common thinking style behind all these variables [12, 21, 23, 24]. We consider approval of paranormal practices to be an indicator of paranormal beliefs. Taking into account the effect sizes presented in the background section, this makes paranormal beliefs and related measures the most important known predictors of the use and approval of CAM.

Our data cannot explain the correlation between the paranormal and CAM. If both originated from intuitive thinking, this would be advantageous in that the properties of intuitive and rational thinking have already been well researched. The known methods of appealing to intuitive thinking or activating rational thinking could help doctors to communicate better with CAM patients. This seems particularly relevant in view of the fact that CAM patients sometimes tend to reject effective therapies, which leads to a worse prognosis in cancer treatment [33]. Traditional religiosity is uncorrelated with CAM in our sample, possibly because Christian theology is too academic to appeal to intuitive thinking. However, if intuitive thinking were the only link between the paranormal and CAM, CAM would have a considerably stronger correlation with intuitive thinking than with paranormal beliefs, which is not what has been empirically found [12, 21].

Another possible connection between the paranormal and CAM could be dualist thinking. Dualist thinking has been shown to be strongly correlated with paranormal beliefs and ontological confusions [34]. In a broader sense, dualist thinking refers to the tendency to think about the world as separated into material and immaterial substances that may interact but exist independently from each other. Many varieties of CAM are based on arguments involving the interaction of mind, spirit, energy, qi or healing information with the body, which we would expect to be most appealing to dualist thinkers. Our own results provide evidence for this hypothesis insofar as belief in reincarnation and belief in life after death, which are prototypical manifestations of dualist thinking, relevantly predict approval of CAM. Further studies should test this hypothesis using validated measures of dualist thinking.

A further explanatory hypothesis is that the appropriation of spiritual explanatory models for diseases enables patients to reframe the disease experiences. This possibly leads to more comprehensibility, controllability and feeling of manageability and thus has salutogenetic significance [35, 36]. Spiritual-religious forms of healing could therefore place the suffering of patients in new contexts of meaning [37]. As presented in the background section, there is some evidence for the connection between CAM and spirituality [8, 20], and moreover, paranormal or supernatural beliefs have been shown to be an aspect of spirituality in previous research [38, 39]. In short, paranormal practices and CAM could stem from the same desire for orientation in life.

We introduced experience with paranormal practices as a new relevant predictor of experience with CAM and demonstrated that this effect was mainly not mediated by approval. Trying CAM or paranormal practices might cause each other somehow, but it seems more likely to us that there are common reasons for trying CAM or paranormal practices, one of which could well be openness to experience.

We found that higher socioeconomic status predicts experience with CAM independently from the opinion of CAM. It is a well-known phenomenon in German outpatient care that people of higher status tend to consult specialists rather than general practitioners [40]. Among the possible explanations discussed in that field of research are differences in health literacy. Health literacy refers not only to the ability but also to the motivation to find and understand information on health and health care providers. Health literacy has been shown to be positively correlated with socioeconomic status in Germany and other European countries [41, 42]. We therefore hypothesize that people of higher status are better informed about CAM services and providers and are more willing to manage additional consultations with and opinions from CAM providers, whereas people of lower status may prefer to “keep things easy” by relying primarily on their general practitioner. Our results speak against financial barriers to experience with CAM, which are still being discussed in the case of specialists’ utilization [40].

We could not fully reproduce the findings of the Swiss study presenting post-materialist values and neo-religious beliefs as relevant predictors of CAM use. We confirmed neo-religious beliefs but rather as an aspect of paranormal belief. Post-materialism, as measured by the Inglehart index, was an independent yet marginally relevant predictor. The questions on important things in life (in particular, respecting law and order, high standard of living and pursuit of security) did not indicate any connection with materialism. This discrepancy may result from differences in the measurement of dependent or independent variables, or it may reflect actual differences between the two populations (members of a Swiss health insurance company vs. cross section of Germany).

Contrary to our expectation, we did not find that bad self-rated health predicts experience with CAM. In another cross-sectional German study conducted in the same year, bad self-rated health increased the odds of having used three or more CAM practices instead of none [6]. However, the effect was small and not clearly significant in view of multiple testing. If illness predicts CAM use, the predictive aspects of illness might be badly represented by self-rated health. The face-to-face design of ALLBUS might have aggravated this problem by keeping the interviewees from complaining. CAM treatments could be more successful in improving self-rated health than in improving the underlying illness. In this case, the correlation between bad self-rated health and CAM use would have to be lower than the correlation between illness and CAM use.

### Limitations

The ALLBUS survey was not designed to answer any particular research question, but to provide representative data that can be used for a variety of research questions. The data set contains hundreds of variables, but no validated measures of CAM belief, paranormal belief or other established constructs. We therefore had to select variables based on how we understand the underlying questions, which might differ from how the interviewees understood them; the ALLBUS authors did not provide definitions. For CAM and paranormal practices, the interviewees had to choose between “I think a lot/a little/nothing at all of it”. We believe this way of asking measures approval. Of course, the interviewees’ attitudes towards CAM and paranormal practices are more complex than this question allowed them to answer. Quantitative research, however, requires a limited set of possible answers.

Use is usually measured as use within the past year. We used lifetime use, which allowed us to consider the interaction with opinion. The disadvantage is that no statement can be made about ongoing use. Unfortunately, ALLBUS does not provide data on individual CAM practices but asked for groups of apparently similar practices. Although the specific bundling seems to be reasonable, we do not have evidence that the items explicitly mentioned are identical or even similar in predictors. Moreover, the extent to which the more general questions on Far Eastern and other alternative therapies have captured CAM practices not explicitly mentioned is not clear. For all these reasons, the fact we did not find differences in predictors does not rule out the possibility of single practices predicted differently. The fact that approval of spiritual healing, which may be the most “esoteric” CAM practice in our analysis, is predicted especially well by approval of paranormal practices could mean that the association is not as strong or inexistent in more “materialist” varieties of CAM such as chiropractic. Nevertheless, the four groups of CAM practices available loaded well on the same dependent canonical variates, which indicates that the dependent canonical variates have captured universal aspects of CAM.

## Conclusions

Paranormal beliefs and related measures are the most important known predictors of the use and approval of CAM. Possible links between the paranormal and CAM include an intuitive or dualist thinking style and a desire for orientation in life. Personal values and worldviews, on the other hand, seem to be a negligible motivation for the use and approval of CAM. Female gender and higher socioeconomic status predict experience with CAM without predicting approval of CAM, but their influence should not be overstated.

## Supplementary information

**Additional file 1.** The unabridged results of the CCA including all 24 unrotated variates, all variables and the canonical correlations.

## Data Availability

The datasets analysed during the current study and a detailed documentation of the survey methodology are available in the GESIS Data Archive, doi:10.4232/1.11753 [28]. The statistical analysis was performed in R (version 3.5.0). In addition to basic functions, we used the following packages: foreign (0.8–70), VIM (4.7.0), and candisc (0.8–0). All this software can be freely downloaded from https://cran.r-project.org/.

## Abbreviations

ALLBUS: Allgemeine Bevölkerungsumfrage der Sozialwissenschaften (English proper name: German General Social Survey)
CAM: Complementary and alternative medicine
CCA: Canonical correlation analysis
